# From Cadaveric Dissection to the Operating Room: A Unilateral Double Intercostobrachial Nerve and the Implications in Axillary Lymph Node Dissection

**DOI:** 10.7759/cureus.36647

**Published:** 2023-03-24

**Authors:** Mariana N Olivencia-Delgado, Javier F Jusino-Álamo, Emanuel De Miranda-Sánchez, Jailenne I Quiñones-Rodríguez

**Affiliations:** 1 Department of Anatomy and Cell Biology, Universidad Central del Caribe School of Medicine, Bayamon, PRI; 2 Department of Surgery, University of Puerto Rico School of Medicine, San Juan, PRI; 3 Department of Clinical Anatomy, Sam Houston State University College of Osteopathic Medicine, Texas, USA

**Keywords:** axillary dissection, neuropathic pain syndrome, alnd: - axillary lymph node dissection, anatomical variations, intercostobrachial nerve

## Abstract

There are multiple treatment options for breast cancer (BC), including lumpectomy, chemo- and radiotherapy, complete mastectomy, and, when indicated, an axillary lymph node dissection. Such node dissections commonly lead the surgeon to encounter the intercostobrachial nerve (ICBN), which, if injured, leads to significant postoperative numbness of the upper arm. To assist in identifying the ICBN, we report a unilateral variation of a dual ICBN. The first ICBN (ICBN I) originates from the second intercostal space, as classically described in human anatomy. On the contrary, the second ICBN (ICBN II) originates from the second and third intercostal spaces. The anatomical knowledge of ICBN origin and its variations are crucial for axillary lymph node dissection in BC and other surgical interventions that involve the axillary region (e.g., regional nerve blocks). An iatrogenic injury of the ICBN has been associated with postoperative pain, paresthesia, and loss of upper extremity sensation in the dermatome supplied by this nerve. Therefore, maintaining the integrity of the ICBN is a worthy goal during axillary dissections in BC patients. Increasing the awareness of ICBN variants among surgeons reduces potential injuries, which would contribute to the BC patient's quality of life.

## Introduction

The intercostobrachial nerve (ICBN) generally originates from the lateral cutaneous branch (LCB) of the second intercostal nerve (ICN) at the level of T2. As the ICBN travels to the medial arm, it pierces the intercostal muscles of the second intercostal space and the serratus anterior in the midaxillary line before it reaches the skin and subcutaneous tissue [1]. As it courses through the axilla, it often joins the medial cutaneous nerve to innervate the arm's medial aspects, the breast's tail, and the lateral chest wall [2,3].

The ICBN is at risk during mastectomies, axillary lymphadenectomies, or any other surgical procedure that involves the axillary region, and severing this nerve has been found to cause postoperative numbness, significant paresthesia, and pain [4]. In general, axillary surgery in breast tumors can damage or lacerate this region's peripheral nerves. Estimates suggest that between 8% and 25.4% of all peripheral nerve injuries are directly caused by medical intervention [5]. Following nerve damage, a cascade of events can occur that comprise alterations in the function of peripheral nerves [6]. These changes may not cause pain but may lead to sensory dysfunction, including hypoesthesia and hyperalgesia [7]. This set of symptoms, which is directly caused by nerve injury, is called neuropathic pain syndrome.

The axillary lymph node dissection involves the removal of all tissue between distinctive anatomical landmarks. Some of these landmarks include the axillary vein, which is located superiorly, the thoracodorsal bundle running laterally, and the chest wall situated medially [8]. Through the dissection, 10-40 lymph nodes are typically removed en bloc [8]. As a result, patients have an increased risk of complications such as lymphedema, limited shoulder motion, and neuropathic pain [8]. The most common neuropathic complication of axillary dissection is intercostobrachial nerve syndrome due to an iatrogenic injury [4]. The present case describes an atypical anatomical variation of the ICBN found in a Puerto Rican elderly cadaver. These findings might impact current awareness of advanced breast surgery by preventing ICBN iatrogenic injury and its clinical complications.

## Case presentation

During routine axillary dissection, an anatomical variation was found in a Puerto Rican elderly female cadaver with a dual ICBN. The clinical history, family history, and cause of death were unavailable. There was no evidence of previous surgical interventions or pathologies concerning the axillary region. 

**Figure 1:**
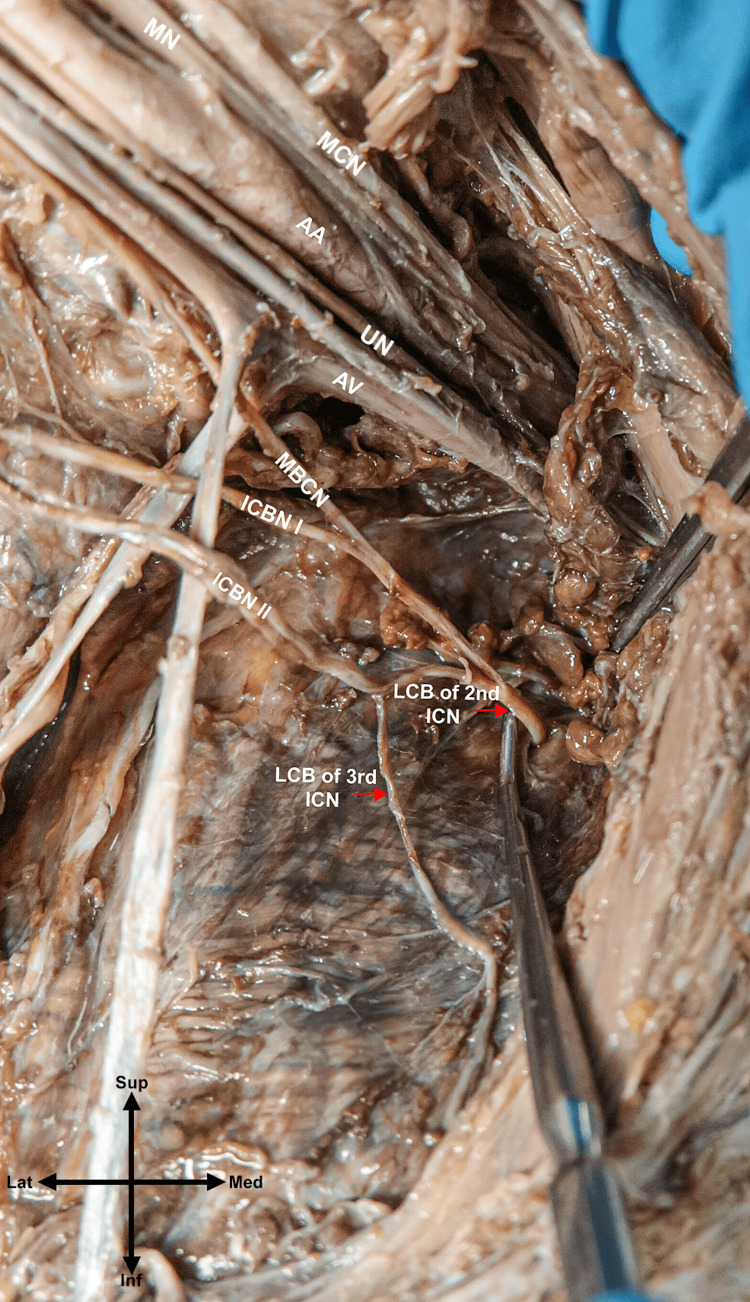
Cadaveric dissection showing the lateral chest wall and axilla with subcutaneous tissue removed The following shows the typically originating intercostobrachial nerve (ICBN) I from the lateral cutaneous branch (LCB) of the 2nd intercostal nerve (ICN) joining the medial brachial cutaneous nerve (MBCN); ICBN II is formed from the LCB of the 2nd ICN and the LCB of the 3rd ICN. Both ICBNs cross inferiorly to the brachial plexus; ICBN I runs posterior, while ICBN II runs anterior to the internal thoracic artery. The general blood supply and nerves of the region were anatomically normal, including the axillary artery (AA), axillary vein (AV), median nerve (MN), the ulnar nerve (UN), and musculocutaneous nerve (MCN).

**Figure 2:**
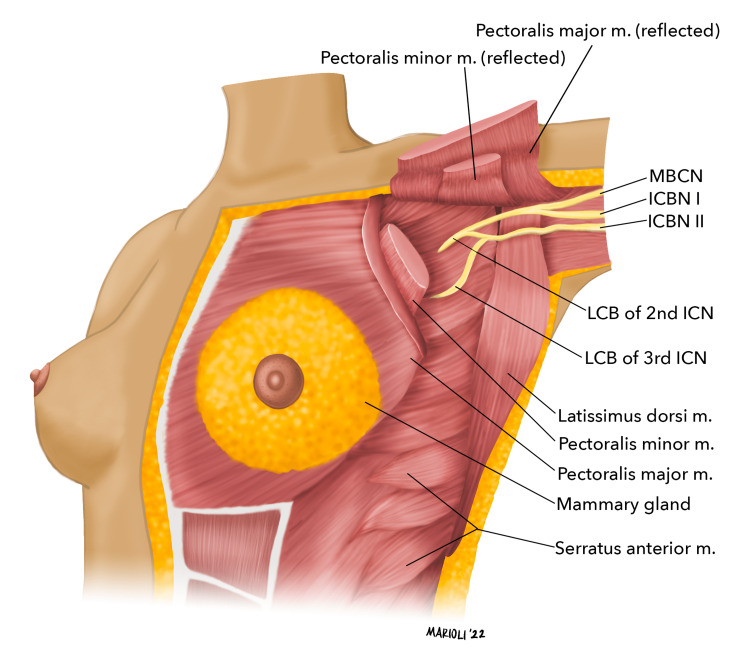
Illustration showing the ICBN variation Illustration showing the reported intercostobrachial nerve (ICBN) variation. The ICBN I, as usual, originates from the lateral cutaneous branch (LCB) of the 2nd intercostal nerve (ICN) and joins the medial brachial cutaneous nerve (MBCN); the atypical ICBN II is formed from the LCB of the 2nd ICN and the LCB of the 3rd ICN. Image credits: Mariana N. Olivencia.

After identifying the anterior surface of the axillary sheath, blunt dissection followed surrounding the axillary neurovascular bundle, and components of the brachial plexus were identified as anatomically usual. The axillary vessels were retracted and identified; coursing classically as described in human anatomy. While exposing the lateral thoracic wall and the contents of the axilla, the ICBN was observed from the second intercostal space bilaterally. However, a unilateral variation in the ICBN was observed from the LCB of the second intercostal nerve joining with the medial brachial cutaneous nerve (MBCN). Specifically, we found a second ICBN (ICBN II) formed from the LCB of the second and third intercostal nerves (Figure 1). Nonetheless, both intercostobrachial nerves (e.g., ICBN I and II) maintained their usual course towards the brachial plexus, extending laterally (Figure 2) and communicating with the posterior and medial cutaneous nerves of the arm to supply the skin of the axilla and medial aspect of the proximal arm. The variation was not observed on the contralateral (left upper extremity) dissection.

## Discussion

The present study aimed to report an ICBN variation formed by the junction of the lateral cutaneous branches from the second and third intercostal nerves. Previous studies have established a high degree of variability in the anatomy of this sensory nerve [9-11]. For instance, Cunnick et al. reported an ICBN variation that arose from the T2 spinal level and bifurcated into a main trunk and a smaller trunk [10]. An additional variant established was an ICBN formed by the junction of one branch from the T1 spinal level and another from the T2 spinal level [10]. Literature also reports ICBN variations in which it communicates with the brachial plexus via the MBCN and the medial cord [11]. Of note, van Tonder et al. described two cases in which the ICBN presented a motor branch for the pectoral muscles [12].

Several studies have reported that the incidence of single-trunk ICBN ranges between 74% and 81.3% [10,13-14]. Another study reported an incidence of 93.3% [15]. Nayak and Banerjee conducted a study in which they identified the origin of the ICBN in 130 axillae; as usual, 100% of the cases presented an ICBN originating from the second intercostal nerve. In addition, they encountered five cases in which this nerve arose from the first intercostal nerve and 27 cases in which it originated from the third intercostal nerve [16]. Similarly, another study reported that, among 156 BC surgery patients, 120 of them had a single trunk ICBN arising from T2 [17].

On the contrary, 23 patients had a double trunk originating from the same level, and nine presented a multiple trunk ICBN [17]. In contrast to the studies above, our donor showed an ICBN originating from the T2 and T3 spinal levels before merging. Surgeons should consider the presentation of this anatomical variation during intervention planning.

From an embryological perspective, the ICBN is derived from the neural crest cells. These cells migrate from the dorsum of the neural tube via epithelial to mesenchymal transition, eventually giving rise to multiple structures, including peripheral nerves (e.g., ICBN). The neural crest cells are highly migratory, and the signaling pathways that lead to their migration are triggered by different transcription factors that promote induction, cell migration, neural migration, and differentiation [18,19]. Thus, we suggest that anomalies in the molecular mechanism of neural crest cell migration might be involved in the abnormal development of upper limb peripheral nerves.

Clinical significance

Moderate to severe postoperative pain is experienced by approximately 50% of patients in the first week after BC surgery [20,21]. While much effort has been put into analgesic regimes to reduce acute postoperative pain, the influence of surgical techniques has been less examined [20]. The ICBN traverses the axilla; therefore, surgeons regularly encounter it during breast cancer (BC) surgical procedures that include axillary lymph node dissections; the ICBN can also be injured during anesthetic nerve blocks in the axillary region, avulsion, and traction injuries to the brachial plexus [16]. An iatrogenic injury to this structure may occur from prolonged pressure from retraction, transection from electrocautery or sharp dissection, and surgical clips [22]. A detailed management plan must be outlined if a patient presents with focal pain on examination after an iatrogenic injury to the ICBN.

Recently, a study demonstrated that patients with the ICBN preserved report less postoperative pain, paresthesia, or loss of sensation in the armpit and inner arm than patients who undergo ICBN injury or transection [23]. La Cesa et al. have noted that sensory disturbances due to ICBN injury improve one year post-BC surgery, but neuropathic pain does not [24]. According to Chirappapha et al., ICBN preservation also benefits the upper limbs' physical function [25]. Nevertheless, one possible limitation of ICBN preservation is the theoretical risk of tumor cell dissemination while carrying out excessive manipulation of the perineural lymphatic vessels and adipose tissue. However, Torresan et al. found no local relapses in BC surgical patients with the ICBN preserved [26]. As such, ICBN preservation can be considered feasible and safe. As a result, preserving this nerve when possible is a worthwhile goal in breast cancer surgical management. Variations such as those reported here can be consistently identified in an anterior position during exposure to the long thoracic and thoracodorsal nerves. On the lateral chest wall, the ICBN could originate from the second intercostal space with fibers from the first or third intercostal spaces, which would be identified as anatomical variations. To better the chances of preserving this nerve, surgeons should be aware of the anatomical variability of the ICBN to avoid its injury and the potential complications associated with it. The present report is a non-described anatomical variation in an elderly Puerto Rican donor of the ICBN. Therefore, reporting this finding would add a possible variant to the previously described ICBN discrepancies.

## Conclusions

In this study, we report an atypical anatomical variation involving the ICBN and the clinical implications it might present. During axillary surgical procedures for breast cancer, the ICBN may be sectioned or injured, potentially leading to postoperative pain, paresthesia, and sensory loss on the medial aspect of the proximal arm. The most effective way to prevent nerve injury during surgery is by identifying the nerve through meticulous dissection. Recognizing the possible ICBN variations would decrease the risk of iatrogenic injury and the consequences that this injury entails for the BC patient.
